# Clinical Evaluation of Microbial Communities and Associated Biofilms with Breast Augmentation Failure

**DOI:** 10.3390/microorganisms12091830

**Published:** 2024-09-04

**Authors:** Robert Whitfield, Craig D. Tipton, Niccole Diaz, Jacob Ancira, Kyle S. Landry

**Affiliations:** 1 Robert Whitfield MD PLLC, Austin, TX 78746, USA; 2 RTL Genomics, MicroGen DX, Lubbock, TX 79424, USAniccole.diaz@microgendx.com (N.D.); jacob.ancira@microgendx.com (J.A.); 3 Department of Health and Rehabilitation Sciences, Boston University, Boston, MA 02215, USA; 4 Delavie Sciences LLC, Worcester, MA 01606, USA

**Keywords:** breast implants, biofilms, inflammation

## Abstract

The incidence of breast implant illness (BII) and BII-related explant procedures has not decreased with current surgical and treatment techniques. It is speculated the main underlying cause of BII complications is the result of chronic, sub-clinical infections residing on and around the implant. The infection, and subsequent biofilm, produce antagonistic compounds that drive chronic inflammation and immune responses. In this study, the microbial communities in over 600 consecutive samples of infected explant capsules and tissues were identified via next-generation sequencing to identify any commonality between samples. The majority of the bacteria identified were Gram-positive, with *Cutibacterium acnes* and *Staphylococcus epidermidis* being the dominant organisms. No correlation between sample richness and implant filling was found. However, there was a significant correlation between sample richness and patient age. Due to the complex nature, breast augmentation failures may be better addressed from a holistic approach than one of limited scope.

## 1. Introduction

Biofilms are combinations of bacterial cells and an extracellular matrix, which is composed of various substances such as proteins, lipids, carbohydrates, and extracellular DNA [1]. Bacterial biofilms impact a wide range of industries and are one of the main targets for various cleaning and sterilization procedures [2,3]. Within the medical field, biofilms are linked to oral dysbiosis, diabetic wounds, surgical complications, respiratory infections, and reactive arthritis [4,5,6,7]. The commercial and consumer goods industry is also plagued by biofilms, causing pipe fouling, food safety recalls. Even equipment malfunctions aboard the International Space Station have fallen victim [8,9,10]. Biofilms can also serve beneficial functions. These include bioremediation, wastewater treatment, agrobiology, and biomanufacturing applications [11,12,13].

With the adaptability and wide-spread nature of bacterial biofilms, their potential impact on the success rate of breast reconstructive surgery should not come as a surprise. Breast reconstruction has multiple applications that range from cosmetic enhancements to reshaping post-tissue loss resulting from cancer. Due to the uplifting physical and psychological impact these procedures can have on the patient population, breast augmentation remains one of the most common plastic surgery procedures performed among women, with over 2 million procedures performed in 2019 alone [14,15]. These procedures do not come without risk [16]. Up to 2.5% of these procedures will have some degree of complications related to infections from microorganisms that result in revising or removal of the prosthesis. The transfer of skin microbiota into the incision or onto the prosthesis can seed the initial infection. The topographical nature of the prosthesis also has an impact. Originally smooth in nature, silicone mammary implants are now available in an array of macrotexture varieties. The macrotexture variants were designed to minimize implant slipping and twisting, which often lead to capsular contractions. However, the textured surface also provides a point of adhesion for bacteria, and thus, biofilm formation [17,18,19]. The cascade of inflammatory events following biofilm formation results in a system-wide response to the prosthesis, which has been shown to accelerate implant degradation and rupture [20,21].

The microbial composition of the biofilm may impact the long-term success rate of breast reconstructive surgery. Multiple genera and species have been identified that are known to be either pathogenic or opportunistic pathogens [22,23]. Frequently isolated organisms include *Staphylococcus epidermidis*, *Staphylococcus aureus*, and *Cutibacterium acnes*, all of which have been associated with chronic inflammation and immune responses [22,24,25]. It should be noted that the frequency, severity, and correlation of infection with specific species or even genus of bacteria are often inconsistent and contradicting.

Unlike traditional bacterial infections, chronic and sub-clinical infections are difficult to identify. Recent studies have shown that up to 48% of patients who have undergone explant surgery for breast implant illness develop a subclinical infection [25]. The researchers also reported that *Cutibacterium acnes*, a biofilm-producing bacteria, was the dominant organism present and a key contributor to chronic inflammation [25].

The following Internal Review Board (IRB)-approved retrospective study analyzed of over 600 consecutive samples of infected explant capsules and tissues to establish the incidence of sub-clinical microbial communities/biofilms and their potential association with breast prosthesis failure

## 2. Materials and Methods

### 2.1. Sample Collection and Institutional Review Board Statement

The chart review was performed from February 2019 to September 2022. All subjects gave their informed consent for inclusion before they participated in this study. This study was conducted in accordance with the Declaration of Helsinki, and the protocol was approved by the Ethics Committee of ADVARRA IRB, CR00487631. On the day of surgery, all the patients underwent capsulectomies, as biospecimens were collected as part of the surgical plan. Capsule tissue removed from the patients was photographed in the operating room by the operating surgeon and sent within 24 h for routine histological analysis. Data collection by the operating surgeon included details about the implant shell (i.e., textured or smooth) and whether the implant was silicone or saline, when available. Changes in the shell of the device were also noted. A piece of capsule and tissue were sent for 16S rRNA gene sequencing at MicroGen DX, Lubbock, TX, USA.

### 2.2. Microbial Profiling

The samples were shipped overnight to MicroGen DX (Lubbock, TX, USA), a CAP-accredited and CLIA-licensed clinical diagnostic laboratory, for microbiological profiling via targeted next-generation sequencing (NGS), similar to previous studies [26,27,28]. Briefly, the commercial assay included two targets for targeted NGS (16s rRNA V1–V2 and ITS3-4) for comprehensive profiling of bacteria. In order for a sample to be sequenced for either target, amplicons were evaluated using endpoint PCR for evidence of positive amplification prior to paired-end 250 base pair sequencing using an Miseq system (Illumina, San Diego, CA, USA). The laboratory-developed test also included a multi-species qPCR panel and multitarget antibiotic resistance gene panel [27,29]. Prior work suggests that although partial 16s analysis cannot fully resolve all bacterial lineages [30], the underlying V1–V2 region used is among the most informative for classifying the species [31,32], and species-level calls are generally reproducible when using appropriately curated databases [33]. The analyses were based on the microbial findings provided in clinical reports, which were reported to the species level where possible, as in previous work.

### 2.3. Statistical Analysis

The patients whose samples were positive were selected for descriptive analysis of the bacteria identified via targeted 16S rRNA profiling. The samples were described in terms of the bacteria detected and summarized according to the number of species (i.e., richness) detected in each specimen. An analysis of variance (ANOVA) was used to screen the available demographic features against richness, with an emphasis on determining whether breast implant filling or texture was related to richness and using backward feature selection to remove non-significant variables from the final model. An additional partial regression model to adjust for confounding factors within the ANOVA was included. A partial regression model was applied in three steps to estimate the relationship of gel filling with species diversity when removing variance confounded by age. First, gel filling was regressed on age and other metadata. Second, species richness was regressed on age and metadata other than gel filling. Third, a final model to estimate the impact of gel filling on species richness independent of age was performed by regressing richness residual variance on gel filling residual variance. Dominant species were determined based on the highest observed species in each patient.

## 3. Results

**Figure 1:**
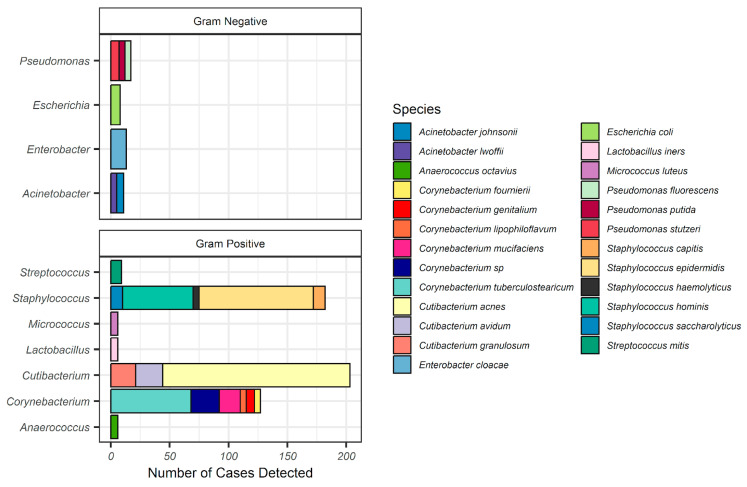
Bar plot showing incidence of the top 25 species, grouped by genus and facetted by Gram staining.

**Figure 2:**
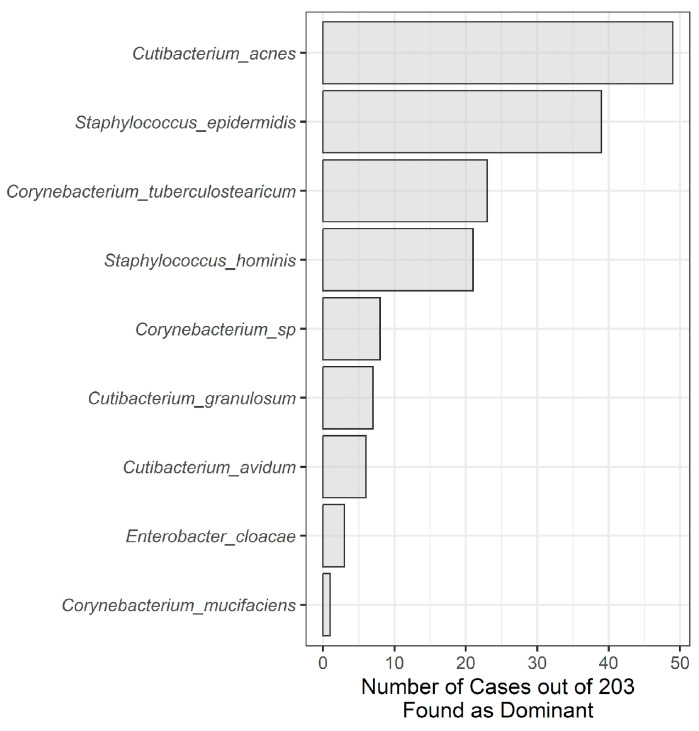
Bar plot of dominant species that occur within at least 5% of samples.

From June 2019 through August 2022, a total of 694 samples were submitted for NGS microbial profiling, and 203 (29%) returned positive microbiological findings. These 203 positive samples were included in the descriptive analysis (see Table 1 for cohort demographics) and revealed 103 unique species. Gram-positive lineages including *Cutibacterium* (formerly *Propionibacterium*), *Staphylococcus*, and *Corynebacterium* were the most frequently detected (Figure 1), though a few Gram-negative lineages were found in 8% and 6% of the samples, such as *Pseudomonas* and *Enterobacter*, respectively. Relatively few species tend to be dominant; however, an emphasis was placed on identifying those species by counting the number of times a species was observed to be dominant (i.e., most abundant species observed). The most dominantly reported species again belonged to the same three genera as before, with *C. acnes*, *S. epidermidis*, and *C. tuberculostearicum* being the most dominant (Figure 2). One Gram-negative species was reported among the most dominant, *Enterobacter cloacae*.

**Figure 3:**
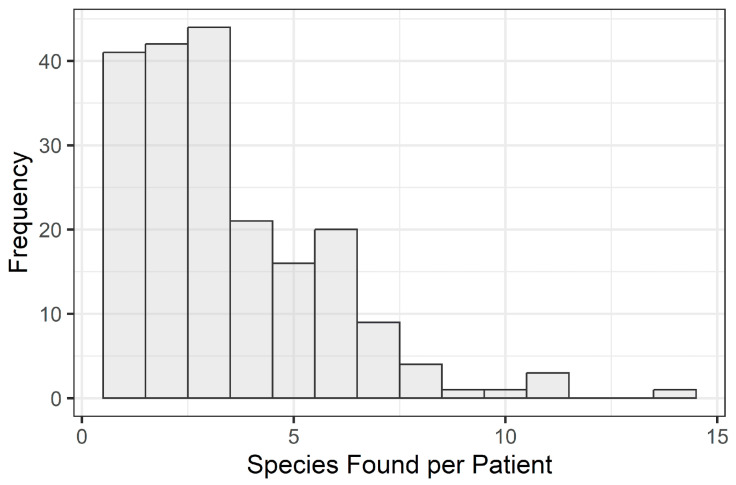
Histogram showing the distribution of sample richness (number of species detected) out of the 203 positive samples.

Next, species richness can be a useful measure for reducing a complex species profile into a single estimate that is sometimes associated with differences in patient characteristics. Here, richness was assessed with the aim of determining whether the type of breast implant had any apparent association with the microbial profile recovered upon removal. A median of 3 species (Q1 = 2, Q3 = 5) were detected, with 72% having fewer than 5 species reported (Figure 3). Implant texture was not found to have any apparent association with richness (Table 2, *p* > 0.05); however, implant filling type and age were associated with richness (*p* < 0.05).

**Figure 4:**
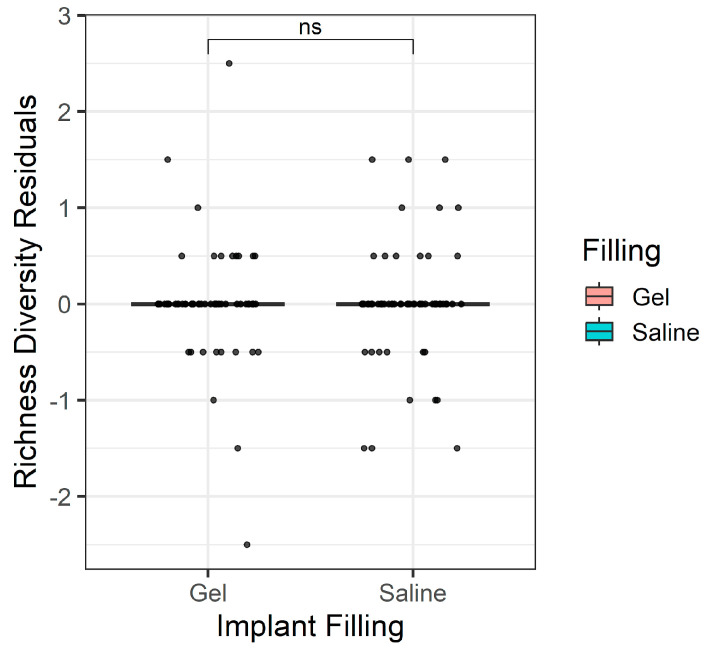
Residual variance plot showing the difference in species richness after controlling for confounding effects of implant filling.

**Figure 5:**
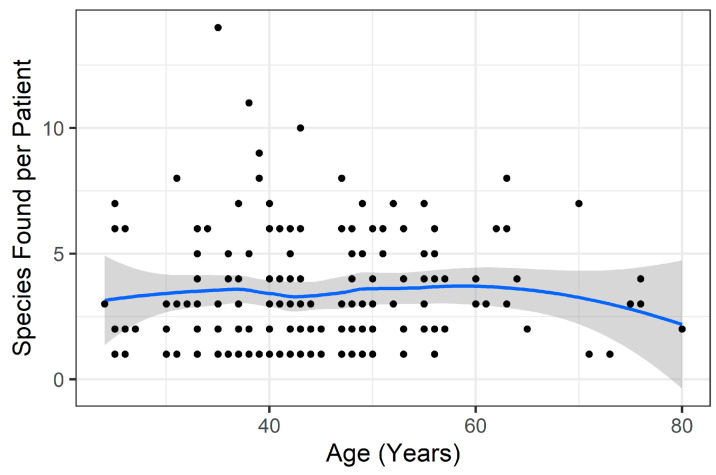
Dot plot showing species richness by patient age. Trend line with confidence interval shown.

However, there was concern that age and filling may be confounded, leading to a spurious association of filling with age. To test this, a partial regression approach was used to first account for the variation explained by age and then to test the importance of filling type against that residual variation, which was no longer statistically significant after accounting for age (*p* > 0.05, Figure 4). In simpler terms, implant filling may be related to the number of species observed, but the statistical association disappears when attempting to control for the confounding influence of patient age. Patient age was the only factor considered significant after all testing (Figure 5).

## 4. Discussion

Bacterial biofilms have an impact on multiple industries, and their dynamic nature makes them difficult to combat. Their robustness is due to the complex extracellular matrix they use to adhere and protect themselves from external threats. This extracellular polymeric substance (EPS) protects vegetative cells from antibiotics, disinfectants, and extraneous organisms by slowing diffusion, the sequestering or inactivation of compounds, and acting as a physical barrier [34,35,36,37]. Enzymatic removal, chemical signaling, bacteriophages, and physical disruption have all been proposed as methods for the elimination and control of bacterial biofilms. The various macromolecules that comprise the EPS matrix, such as carbohydrates, nucleic acids, proteins, dead bug bodies, etc., are often the target for the enzymatic removal of biofilms. Hydrolyzing the key structural components, in theory, would help with the removal of biofilms. Various nucleases, lipases, proteases, and carbohydrases have been studied; however, the use of enzymes is often limited to treating surfaces and equipment, and their potential use for treating implant infections is negligible [38,39,40,41,42]. The same can be said for the use of bacteriophages. Though quite successful for surfaces and food, the use of viruses for the successful treatment or mitigation of biofilms related to breast implants has yet to be demonstrated [43,44,45].

The dominant organisms found in this reflective study align with previous findings; however, the sample size presented is significantly larger than most previous studies, which allows for deeper insights into a complex problem. As highlighted in Figure 3, the microbial richness was small, with most patients having fewer than five species. Though this has only identified the presence of the microbiota, the correlation between the species identified and their role in biofilm-related breast augmentation failure has been well established in the literature [46,47,48,49,50,51]. No correlation between fill type was found; however, patient age did have an impact on diversity. It is well established that the skin microbiome changes over the course of our lives. For example, the maturation of an infant’s microbiome starts at birth with the mother’s vaginal microbiome during a vaginal birth or a combination of skin and environmental bacteria during a cesarean birth [52,53]. From there on, external factors drive microbiome diversification. As children progress through adolescence and into adulthood, hormonal factors start to influence sebum production on the skin. This results in a dramatic shift in the skin microbiome. Once predominantly Firmicutes, Bacteroidetes, and Proteobacteria, the increased sebum levels drive the proliferation of *Cutibacterium acnes* and *Staphylococcus epidermidis* [54,55,56,57,58]. The rapid growth often results in skin dysbiosis, acne, and an increase in inflammation. Depending on a person’s skin type, lifestyle, and overall well-being, the impact of the shift may be lifelong. This may shed light on why *Cutibacterium acnes* and *Staphylococcus epidermidis*, both of which are known to form robust biofilms in vivo, were the most prevalent organisms found in this study.

Due to their complex nature, bacterial biofilms continue to plague the medical field, including both cosmetic and reconstructive breast augmentation. Biofilm proliferation, and the innate immune response triggered by it, has been associated with capsular contracture, subclinical infection, and breast implant-associated anaplastic large cell lymphoma (BIA-ALCL) [59,60,61,62]. Attempts to prevent bacterial biofilm formation and subsequent infections using antibiotic washes and acidic solutions have been unsuccessful [63,64,65,66,67]. These methods are only effective at the time of insertion of breast implant(s) and offer no protection during the lifetime of the implant from subsequent bacterial exposure. A simple break in the skin barrier creates a bacterial entry point that can cause an immune response at the implant site or drive biofilm formation, with or without the development of cellulitis [68,69,70]. This was demonstrated by two recent randomized prospective studies that highlighted that 48% and 29% of infected implants had measurable biofilms [71,72,73]. In both studies, the predominant organism was *Cutibacterium acnes*; however, other biofilm-related organisms such as *Staphylococcus epidermidis* and *Enterobacter* spp. were also prevalent. Additionally, a recently published study found that breast implant illness (BII) patients who have advanced systemic symptoms were shown to have an increased abundance of biofilm biomass and elevated levels of oxylipin 10-HOME [74]. Oxylipin 10-HOME is a unique fatty acid metabolite that is produced from bacterial biofilms [74,75]. Interestingly, this compound was found to accumulate on the surface of implants and elicited an immune response to the implant and surrounding tissues [74]. These findings demonstrate how subclinical infections drive immune response and prosthesis degradation. Yet, this would suggest that the surgical removal of the entire scar capsule and the device would alleviate BII in its entirety. Additionally, follow-up treatments options would focus on restoring the immune function of each patient. The inherited ability to process toxin exposure is individualized; therefore, a personalized approach must be taken [76,77,78]. Though this is known, it is still unclear when one’s genetic ability to process toxins has reached a saturation point [76]. For example, a single-nucleotide polymorphism (SNP) in the methylenetetrahydrofolate reductase gene (MTHFR) can lead to prolonged inflammation and exacerbate toxin interactions [79,80]. A host of genes involved in methylation and cellular detox processes in both animals and plants are often suppressed in the presence of toxins, and their overall impact on the detoxification process is reduced [81,82,83,84].

There have been numerous studies exploring how to minimize the incidence of biofilm formation and capsular contraction, yet no effective solution has been found. A retrospective cohort study comparing 27 patients who received a triple-antibiotic pocket irrigation containing cefazolin, gentamicin, and bacitracin showed the capsular contraction rate was the same as the 28-patient control group [85,86]. Another group showed that antibiotic irrigation had a significant impact on capsular contraction on both textured and smooth implants [87]. Outside of antibiotics, the use of povidone–iodine is another option. A retrospective study including 3002 patients showed that the use of povidone–iodine significantly reduced the risk of infection following breast augmentation surgery [88]. The results of that trial were not surprising, since a randomized, double-blinded trial performed in 1986 showed a similar outcome [89]. The incidence of capsular contraction was reduced from 41% to 18% with a instillation of a 5% povidone–iodine solution [89]. The lack of consistency around antibiotic irrigation has resulted in the use of povidone–iodine and triple-antibiotics for both pocket irrigation and for soaking breast implants pre-surgery [90,91,92]. Out of all the techniques used to minimize the incidence of capsular contraction, the non-touch funnel has proven impactful [93,94]. Minimizing the number of contact points with the implant significantly reduces microbial transfer, and when combined with pocket pH treatments, it appreciably increases the success rate of an implant procedure [95,96].

It should not go unnoticed that lifestyle and overall wellbeing have been shown to significantly impact the skin microbiome and the body’s ability to respond to inflammation [97,98]. A sub-clinical persistent localized bacterial infection induces a chronic inflammatory response that could have a cumulative negative impact systemically [99]. The ability to control or mitigate the results is dependent on genetic predisposition and intentional choices made throughout a lifetime. One such choice that is often disregarded as a driver of skin dysbiosis and systemic inflammation is diet. There is strong evidence that diet impacts hormone balance and sebum production, both of which impact skin dysbiosis and inflammation [100,101,102]. The “Western diet”, high in red meats and ultra-processed foods, has specifically been shown to negatively impact the skin and gut microbiome while further promoting inflammation and dysbiosis of the gut [103,104,105,106,107]. Overall, there is a strong correlation between microbial communities/biofilms, chronic inflammation, and implant failures. However, the role of external drivers of those key points, such as diet, lifestyle, genetic predisposition, and overall approach to wellness, is often not included in the equation. The persistence of breast augmentation failures may be better addressed from a holistic approach than one of limited scope.

## Figures and Tables

**Table 1:** Specimen characteristics of the samples taken.

Characteristic	N = 203
Median age	43 (Q1 = 37, Q3 = 50)
Left capsule	103 (51%)
**Implant texture**	
Smooth	60 (71%)
Textured	25 (29%)
Missing	118
**Implant filling**	
Gel	65 (49%)
Saline	67 (51%)
Missing	71
Ruptured	13 (6.4%)

**Table 2:** ANOVA assessing relationship sbetween specimen characteristics and species richness.

	Df	Sum Sq	Mean Sq	f-Value	*p*-Value	R^2^	Sig
Texture	1	0.90	0.90	0.56	0.465	0.003	
Filling	1	9.26	9.26	5.75	0.028	0.027	*
Age	1	17.65	17.65	10.96	0.004	0.051	**
Patient	57	291.55	5.11	3.17	0.004	0.837	**
Residuals	18	29.00	1.61				

Df = Degrees of freedom; * denotes significance with *p* < 0.05; ** denotes significance with *p* < 0.01.

## Data Availability

The original contributions presented in the study are included in the article, further inquiries can be directed to the corresponding authors.
